# Impact of Third-Generation Cephalosporin Resistance on Recurrence in Children with Febrile Urinary Tract Infections

**DOI:** 10.3390/jpm12050773

**Published:** 2022-05-10

**Authors:** Sin Young Kim, Min Sik Jang, Jihye Kim

**Affiliations:** Division of Infectious Diseases, Department of Pediatrics, Hallym University College of Medicine, Kangdong Sacred Heart Hospital, Seoul 05355, Korea; 210105@kdh.or.kr (S.Y.K.); 190342@kdh.or.kr (M.S.J.)

**Keywords:** urinary tract infection, third-generation cephalosporin resistance, vesicoureteral reflux, recurrence

## Abstract

Background: The purpose of this study was to investigate the association between third-generation cephalosporin resistance and urinary tract infection (UTI) recurrence in patients who underwent voiding cystourethrogram (VCUG). Methods: In this retrospective study, data were obtained from hospitalized pediatric patients who had a first febrile UTI episode and subsequently underwent VCUG. Information based on VCUG was mandatory to identify the presence of vesicoureteral reflux (VUR). A multivariable logistic model was used to identify the risk factors for recurrence. Recurrence was divided into early (90-day) and late (1-year), and sensitivity analyses were performed according to each definition. The estimates of all the statistical models were internally validated using bootstrap samples. Results: A total of 210 patients were included, and the overall recurrence rate of UTI was 26.2% (55 of 210). Third-generation cephalosporin resistance was a significant risk factor for early recurrence (odds ratio: 2.79 [1.08–7.20]) but not for late recurrence. Sensitivity analyses showed that third-generation cephalosporin resistance was a significant risk factor for 60-day recurrence but not for 180-day recurrence. A VUR grade ≥ 3 was identified as a consistent risk factor for both early and late recurrence. Conclusions: Third-generation cephalosporin resistance was a significant risk factor for the early recurrence of pediatric UTI in patients who underwent VCUG.

## 1. Introduction

Urinary tract infection (UTI) is the most frequent cause of occult and serious bacterial infection in children [1]. Among common UTI pathogens, third-generation cephalosporin-resistant *Enterobacteriaceae* have been increasing in children [2]. Moreover, the results from culture and susceptibility testing are often unavailable until 48 h after initial presentation; such increasing incidence of resistant organisms in community-onset pediatric UTIs affects the appropriate selection of initial empiric treatment in these patients. Tracking trends in local pathogen resistance over time and assessing their impact are essential to develop adequate empiric treatment protocols and antibiotic stewardship programs. 

A substantial increase in the prevalence of community-acquired UTIs with antibiotic resistance among children is recognized globally due to widespread empiric broad-spectrum β-lactam use, creating selective pressure [3,4,5,6]. Due to the limitations and variability of testing and reporting practices across laboratories, third-generation cephalosporin resistance is often used as a surrogate marker of organisms that produce extended-spectrum β-lactamase (ESBL) and other β-lactamases [2,7]. The production of ESBL from plasmid-encoded resistance genes, such as CTX-M, is the principal mechanism underlying resistance in third-generation cephalosporin-resistant *Enterobacteriaceae* [8,9]. These resistant organisms produce variants of the TEM, SHV, and CTX-M β-lactamases and are resistant to all β-lactam antibiotics, except carbapenems [10,11]. Pediatric UTIs caused by these organisms are of concern because the usual first-line therapeutic choices are ineffective in vitro, and no specific guidelines exist for the treatment of third-generation cephalosporin-resistant UTIs.

Although the isolation of *Enterobacteriaceae* that are resistant to third-generation cephalosporins potentially limits treatment options in pediatric UTIs, these patients are often treated with non-susceptible antibiotics. Few studies have described the association of third-generation cephalosporin resistance with future outcomes or recurrence rates [12,13,14]. As vesicoureteral reflux (VUR)—a condition present in approximately 30% to 50% of children with a history of one or more UTIs [1]—is a well-known risk factor for recurrent UTIs [7], the presence of VUR may act as a confounding factor while identifying risk factors for recurrent UTIs. The purpose of this study was to investigate the clinical impact of third-generation cephalosporin resistance on the recurrence of pediatric UTIs, regarding the status of VUR.

## 2. Methods

### 2.1. Study Design and Ethical Considerations

This retrospective study included consecutive hospitalized patients aged 18 years or younger who were diagnosed with a first febrile UTI episode between January 2011 and December 2019. This study was designed and conducted using the format recommended by the Strengthening the Reporting of Observational Studies in Epidemiology guidelines and approved by the Institutional Review Board of Kangdong Sacred Heart Hospital. The Institutional Review Board waived the requirement for informed consent for this study.

### 2.2. Study Cohort

Patients with their first febrile UTI caused by a single *Enterobacteriaceae* organism were included. Afebrile patients or patients with apparent sources of fever other than UTI were excluded. Patients who had received antibiotics prior to hospitalization or those with a history of previous UTI were also excluded. If more than one episode of pediatric febrile UTI was reported for the same patient, only the first episode was included in the study. Since changes in the empiric antibiotic regimen during treatment could influence the association between third-generation cephalosporin resistance and recurrence, such patients were excluded. Patients who subsequently underwent radiologic examinations, including renal ultrasonography (US), ^99^Tc-labeled dimercaptosuccinic acid (DMSA) renal scan, and voiding cystourethrogram (VCUG) were included. A minimum one-year follow-up after the first UTI episode was mandatory for study inclusion.

### 2.3. Definitions

We defined febrile UTI in patients with both (1) pyuria on urinalysis or urine dipstick (≥5 white blood cells per high-power field (centrifuged specimen) or ≥1 leukocyte esterase on dipstick) and (2) urine culture of a single organism with ≥10^5^ colony-forming units (CFUs)/mL for midstream clean-catch collection, ≥5 × 10^4^ CFUs/mL for catheterization, or any bacterial growth for suprapubic collection [1,15]. Urine samples were obtained using suprapubic aspiration, bladder catheterization, or the clean-catch method (considered positive when two or more repeated urine specimens within 24 h met the criteria for UTI) for non-toilet-trained children and using the clean-catch method for old toilet-trained children. Identification and susceptibility testing of the microorganisms was performed using the MicroScan WalkAway 96 plus automated ID/AST system (Beckman Coulter, Inc., Brea, CA, USA). Antibiotic susceptibilities were determined according to Clinical and Laboratory Standards Institute guidelines [16].

Fever was defined as a documented temperature of at least 38 °C or higher within 24 h before or after urine collection. Clinical response was defined as the resolution of fever and the initial symptoms. Bacterial eradication was defined as the absence of the causative pathogen in repeated urine culture after 48–72 h of antibiotic therapy. Third-generation cephalosporin resistance was defined as non-susceptibility to ceftazidime, ceftriaxone, or cefotaxime. Recurrent UTI was defined as subsequent episodes of febrile UTIs after the eradication of the causative organism and termination of therapy for the previous UTI [17]. Recurrence was divided into early (90-day) and late (1-year) recurrence.

### 2.4. Management Protocol

Patients diagnosed with UTIs were initially started on empiric antibiotics and subsequently tailored according to their clinical responses and antibiogram. Antibiotic treatment was administered intravenously, and the patients were switched to oral antibiotics at discharge. Patients who had completed the total duration of therapy with intravenous antibiotics exclusively were discharged without medication. The empiric antibiotics administered were as follows: ampicillin-sulbactam, ampicillin-sulbactam combined with an aminoglycoside, ampicillin-sulbactam combined with a third-generation cephalosporin, a third-generation cephalosporin, or a third-generation cephalosporin combined with an aminoglycoside. The total duration of antibiotic therapy, including the oral regimen, was 10–14 days. Urine culture was repeated after 48–72 h of antibiotic therapy, and if urine did not become sterile, this was repeated until bacterial eradication was achieved. If the urine became sterile during therapy, no further urine culture was performed after the completion of antimicrobial treatment. If the initial antibiotic susceptibility of the patient showed third-generation cephalosporin resistance while the patient responded clinically and microbiologically during treatment with non-susceptible antibiotics, treatment was not modified. If the patient showed a poor clinical response in the presence of third-generation cephalosporin resistance, switching to or the addition of an aminoglycoside (amikacin) or modification to either piperacillin-tazobactam or ertapenem were considered.

### 2.5. Protocol for Renal Imaging Studies

Patients underwent renal US and ^99^Tc DMSA renal scans within 5 days of admission (acute phase). VCUG was performed during hospitalization after the sterilization of urine according to standard guidelines and clinicians’ judgments, if US revealed hydronephrosis, scarring, or other findings suggesting obstructive uropathy, as well as atypical clinical circumstances [1]. Renal parenchymal defects and hydronephrosis were recorded on US. Two nuclear medicine pediatric specialists independently interpreted DMSA scans. Urinary tract anomalies included anatomical abnormalities other than VUR or hydronephrosis (for example, ureterocele, renal agenesis, renal dysplasia, ureteropelvic junction obstruction, ectopic ureter, and horseshoe kidney). VUR was graded according to the 5-grade system of the International Reflux Study Group [18].

### 2.6. Covariates

Data on patient demographics, recent medical history (any hospitalization for >2 days or antimicrobial therapy lasting >48 h within the 6 months preceding admission), and clinical characteristics (age, sex, the presence of fever over 39 °C, the duration of fever at admission, time to fever resolution, and the length of hospital stay) were retrieved from their medical records. Laboratory data included the complete blood cell count, C-reactive protein level, and erythrocyte sedimentation rate. Microbiologic information on species identification and susceptibility and the results of follow-up urine cultures were investigated. Radiologic data based on renal US, acute phase ^99^Tc DMSA renal scans, and VCUG were also included.

### 2.7. Statistical Analysis

Power and sample-size calculations based on a previous report [13] indicated that 159 patients with UTIs were required to have 95% power to detect an odds ratio (OR) of 3.3, assuming a 13.8% recurrence rate and type I error of 0.05. Data were compared using an independent *t*-test for numerical variables and chi-square or Fisher’s exact tests for categorical variables.

Logistic regression analysis was performed to identify risk factors for early and late recurrence. A multivariable adjustment was performed for independent variables identified as significant (*p* < 0.05) in the univariate analysis. Multicollinearity between covariates was tested using the variance inflation factor, and a calibration of the multivariable model was assessed using Hosmer–Lemeshow goodness-of-fit statistics. The estimates of the multivariable model were internally validated with a relative bias on 1000 bootstrapped samples. Relative bias was estimated as the difference between the mean bootstrapped parameter estimates, and the mean parameter estimates of the multivariable model divided by the mean parameter estimates of the multivariable model. Recurrence-free survival was compared according to the risk factors and presented as survival probability and 95% confidence intervals (CIs) using the Hosmer–Lemeshow test. In addition, a sensitivity analysis was performed using shorter (60 days after diagnosis) and longer (180 days after diagnosis) time-based definitions of early recurrence. All analyses were performed using SPSS (version 25.0; IBM Corp., Armonk, NY, USA).

## 3. Results

### 3.1. Baseline Demographic and Clinical Characteristics

**Figure 1:**
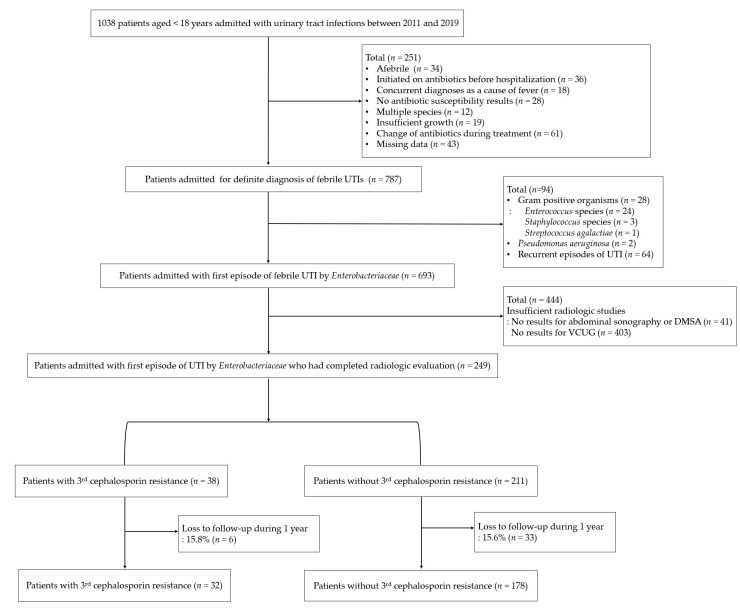
Flow diagram of the study process.

A total of 210 children were finally included in the analysis after applying the exclusion criteria (Figure 1). The baseline characteristics of the entire cohort of patients and those of the two subgroups with or without 1-year recurrence are presented in Table 1. The majority (71.0%) of patients were male, with a median age of 0.4 years (interquartile range: 0.3–0.7). The overall rate of third-generation cephalosporin resistance was 15.2% (32/210). The most frequently identified pathogens from urine cultures were *Escherichia coli* (83.8%, 176/210), followed by *Klebsiella* species (7.6%, 16/210), *Enterobacter* species (5.2%, 11/210), *Proteus mirabilis* (1.0%, 2/210), *Citrobacter* species (1.0%, 2/210), and others (1.5%, 3/210). VUR of any grade and a grade ≥3 was diagnosed in 33.3% (70/210) and 23.8% (50/210) of patients, respectively.

Empiric parenteral antibiotic treatment was as follows: third-generation cephalosporin (55.7%, 117/210), ampicillin-sulbactam and aminoglycoside (27.6%, 58/210), ampicillin-sulbactam and third-generation cephalosporin (11.0%, 23/210), third-generation cephalosporin and aminoglycoside (4.8%, 10/210), and ampicillin-sulbactam only (1.0%, 2/210). Oral antibiotics at discharge were as follows: third-generation cephalosporin (83.3%, 175/210), trimethoprim-sulfamethoxazole (3.8%, 8/210), amoxicillin-clavulanate (3.8%, 8/210), first or second-generation cephalosporins (1.0%, 2/210), and none (8.1%, 17/210), in which treatment was completed with intravenous antibiotics exclusively (Supplementary Table S1).

### 3.2. Antibiotic Susceptibilities

Antibiotic susceptibilities in the urinary isolates of the whole cohort, of the patients with third-generation cephalosporin resistance, and of the initial episodes of the patients with recurrence are shown in Supplementary Table S2. Among the 55 patients with recurrence, 80.0% (8 of 10) and 68.9% (31 of 45) of the patients with or without third-generation cephalosporin resistance experienced recurrence of the same species with the same resistance pattern, respectively.

### 3.3. Risk Factors for Early and Late Recurrence of UTIs: A Multivariable Analysis

**Figure 2:**
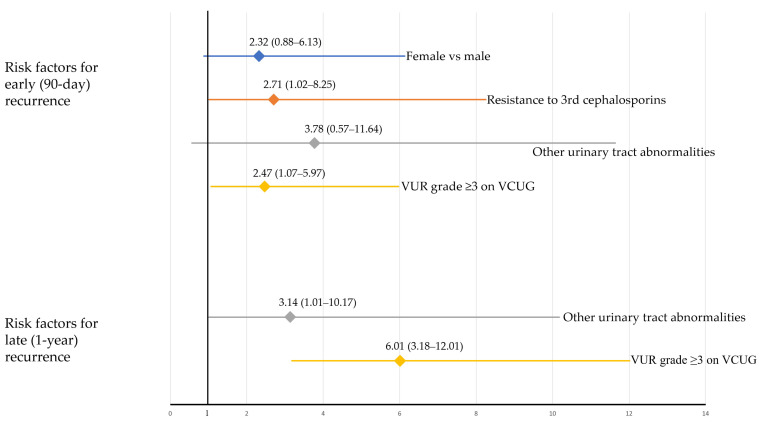
Bootstrap-adjusted odds ratios of various risk factors for early (90-day) and late (1-year) recurrence.

The multivariate analysis identified female sex (OR: 2.37 [1.03–5.44], *p* = 0.042), third-generation cephalosporin resistance (OR: 2.79 [1.08–7.20], *p* = 0.034), and VUR grade ≥ 3 on VCUG (OR: 2.45 [1.04–5.81], *p* = 0.041) as risk factors for early recurrence (Table 2). In contrast, a VUR grade ≥ 3 on VCUG (OR: 6.06 [3.00–12.25], *p* < 0.001) was the only risk factor for late recurrence. Multicollinearity among the independent variables was low, and all the variance inflation factors were <1.5. The Hosmer–Lemeshow goodness-of-fit test indicated good calibration (*p* = 0.554 for early recurrence; *p* = 0.364 for late recurrence). After the bootstrap adjustment, the relative biases of estimates on the multivariable model were low for early (2.2% for female sex, 3.0% for third-generation cephalosporin resistance, and −0.7% for VUR grade ≥ 3) and late (0.6% for a VUR grade ≥ 3) recurrence. Bootstrap-adjusted ORs and their 95% CIs are presented in Figure 2.

### 3.4. Recurrence-Free Survival According to VUR (Grade ≥ 3) and Third-Generation Cephalosporin Resistance

**Figure 3:**
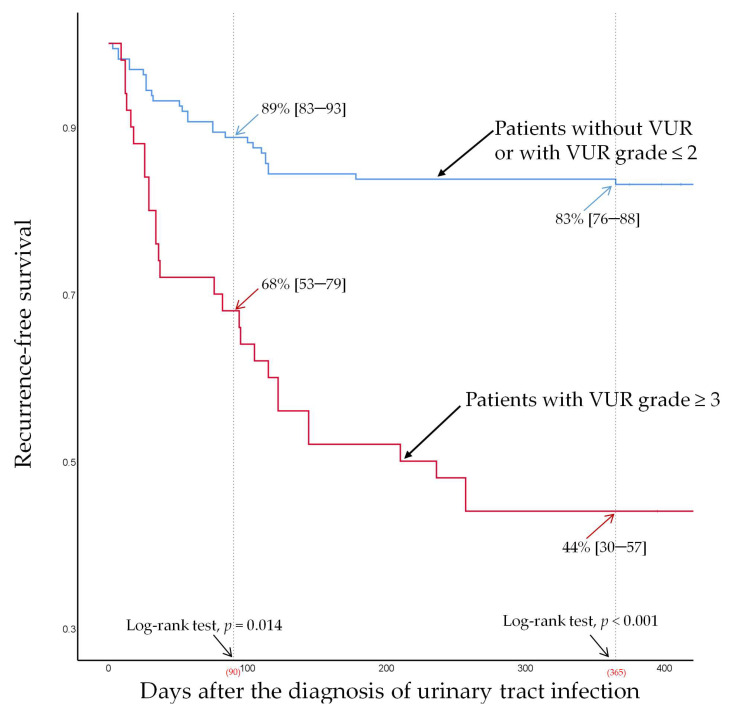
Recurrence-free survival for patients with or without a vesicoureteral reflux grade ≥ 3.

**Figure 4:**
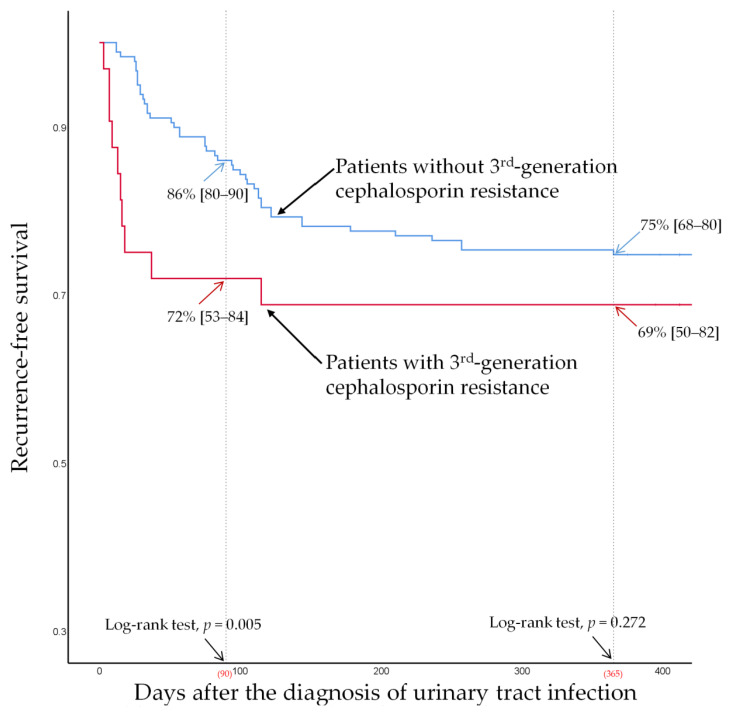
Recurrence-free survival for patients with or without third-generation cephalosporin resistance.

At a 90-day follow-up (for early recurrence), the recurrence-free survival was 68% (95% CI [53–79]) and 89% (95% CI [83–93]) in patients with and without a VUR grade ≥ 3, respectively, and the intergroup differences were significant (log-rank test, *p* = 0.014; Figure 3). The recurrence-free survival was 72% (95% CI [53–84]) and 86% (95% CI [80–90]) in patients with and without third-generation cephalosporin resistance, respectively, and the intergroup differences were also statistically significant (log-rank test, *p* = 0.005; Figure 4) at the 90-day follow-up.

At a 1-year follow-up (for late recurrence), the recurrence-free survival was 44% (95% CI [30–57]) and 83% (95% CI [76–88]) in patients with and without VUR grade ≥ 3, respectively, and the intergroup difference was statistically significant (log-rank test, *p* < 0.001; Figure 3). However, recurrence-free survival was not significantly different (log-rank test, *p* = 0.272, Figure 4) between patients with third-generation cephalosporin resistance (69%, 95% CI [50–82]) and those without resistance (75%, 95% CI [68–80]) at 1-year follow-up.

### 3.5. Sensitivity Analysis 

Sensitivity analysis was performed to assess the multivariable model for early recurrence, using shorter (60-day) and longer (180-day) time points for the definition of early recurrence (Supplementary Tables S3–S6). When an early recurrence was defined as a recurrence within 60 days after the diagnosis, third-generation cephalosporin resistance (OR: 3.15 [1.09–9.11], *p* = 0.034) and VUR grade ≥ 3 on VCUG (OR: 3.93 [1.55–10.02], *p* = 0.004) remained significant risk factors for 60-day recurrence. However, when an early recurrence was defined as a recurrence within 180 days after the diagnosis, third-generation cephalosporin resistance was not a significant risk factor.

## 4. Discussion

In this retrospective study of 210 hospitalized children with febrile UTIs by *Enterobacteriaceae* who had completed radiologic studies including the renal US, ^99^Tc DMSA renal scan, and VCUG, we found that third-generation cephalosporin resistance of the causative organism at the initial episode did not affect the overall recurrence rate at the 1-year follow-up. However, the recurrence-free survival analysis showed that, despite similar rates of 1-year recurrence, patients with third-generation cephalosporin resistance in our cohort had a higher rate of early recurrence than those without cephalosporin resistance (Figure 4). The multivariable analysis identified third-generation cephalosporin resistance as a significant risk factor for 90-day recurrence (OR: 2.79 [1.08–7.20], *p* = 0.034; Table 2). Sensitivity analysis showed that third-generation cephalosporin resistance was a significant risk factor for 60-day recurrence but not for 180-day recurrence (Supplementary Tables S3–S6). A VUR grade ≥ 3 was identified as a consistent risk factor for both early and late recurrence.

In prior studies, risk factors associated with recurrent UTIs were race, age, VUR, renal scarring at baseline, and bladder/bowel dysfunction [17,19,20]. VUR is a well-known risk factor but a weak predictor of renal parenchymal defects [21], and a major risk factor for recurrence [17,18,20]. For studies investigating risk factors for recurrent UTIs, the presence of VUR should be confirmed to minimize confounding effects due to the unknown status of VUR. Therefore, we excluded patients without VCUG results, although new guidelines support restrictive and targeted imaging strategies after the initial infection [1,18]. To the best of our knowledge, this is the first study to investigate the association between UTI recurrence and third-generation cephalosporin resistance, adjusting for VUR status. 

Studies on third-generation cephalosporin resistance in pediatric UTIs have mainly focused on their predisposing risk factors, clinical features, and treatments. The receipt of UTI antimicrobial prophylaxis [3,22], recent hospitalization [3,12,13,23], pre-existing neurological diseases [6], or other underlying diseases [14,22,24] have been reported as risk factors for UTIs due to ESBL-producing microorganisms. Furthermore, discordant antibiotic therapy was common [25] with longer hospital stays [12,13,26], but time to fever resolution was not different [26,27,28]. 

Only a few studies have been published on the association between antibiotic resistance and future recurrences in pediatric UTIs, which have yielded inconclusive results. A high rate of recurrence within 30 days of the initial episode was observed in third-generation cephalosporin-resistant UTIs in a retrospective study [13], whereas other studies have observed no difference in relapse rates [14,29,30]. Some studies have included outpatients or non-febrile UTIs and assessed relapse rates defined as UTI by the same pathogen within 2 weeks after treatment [14,25,29]. In our study, however, we included hospitalized patients with febrile UTIs and focused on early recurrence within 90 days of initial treatment. Thus, recurrence rates based on different definitions, different antibiotic regimens, different settings, a small number of recurrent cases, and no adjustment for VUR may have led to varied conclusions. In addition, these studies lacked mandatory follow-up periods and presented data based on the univariable analysis.

We found that in our cohort, the early recurrence rate (at 90 days) was higher in patients with third-generation cephalosporin resistance than in those without resistance. The reasons for this must be inferred. First, it is possible that patients with antibiotic resistance have higher baseline risk factors for UTI. Although the two groups had similar predisposing risks for subsequent recurrent UTIs, such as urinary tract anomalies (6.3% in the resistant group vs. 7.3% in the non-resistant group, *p* = 1.000) and VUR (37.5% in the resistant group vs. 32.6% in the non-resistant group, *p* = 0.587), other unidentified confounding factors could have affected recurrence rates. Treatment with non-susceptible antibiotics in pediatric UTIs caused by resistant organisms have shown favorable clinical effectiveness in previous studies [25,28,29,30]. However, all patients with third-generation cephalosporin resistance in our study were empirically treated with non-carbapenem antibiotics, and the majority (84.4%) of them with non-susceptible antibiotics, which might have influenced the outcome. Next, according to the protocol of our institution, patients initially treated with non-susceptible antibiotics who improved clinically and microbiologically were discharged with oral antibiotic therapy. The favorable clinical efficacy of discordant antibiotics, despite nonsusceptibility in vitro, is usually explained by the pharmacodynamic/pharmacokinetic goals achieved in the urinary tract [31,32]. Such high concentrations might not be achieved with orally administered antimicrobial agents, leading to incomplete eradication.

The results of our study suggest the following strategies to prevent early recurrence in patients with third-generation cephalosporin resistance. First, initial empiric antibiotic choice and duration may follow the current guidelines, although it should be individually tailored by associated urologic anomalies or the presence of a VUR grade ≥ 3. However, in patients with third-generation cephalosporin resistance and these coexisting conditions, a longer duration of parenteral therapy or discharge with oral antibiotics other than third-generation cephalosporins may be suggested. Second, a close observation of symptoms with a regular follow-up of urine cultures for the detection of the early phase of recurrent UTIs can be suggested. Third, a focused therapeutic approach should be considered in selected patients who have multiple risk factors for early recurrence (combined third-generation cephalosporin resistance and a VUR grade ≥ 3). Susceptible antibiotics, such as carbapenems, can be cautiously suggested in patients with recurrent UTI who have these risk factors. On the contrary, for patients without these risk factors, the current practice of maintaining the initial regimen would be reasonable if the patient showed clinical and microbiological responses while on empiric antibiotic therapy to which the causative bacterium is not susceptible in vitro.

Several limitations affect the interpretation of this study. First, a small number of patients were included in the study. To overcome this limitation, we internally validated our multivariable model using bootstrapped samples, and the relative bias of our multivariable model calculated by the bootstrap method was low. In addition, we also performed a sensitivity analysis according to different time points for early recurrence and confirmed an increased rate of early recurrence in patients with third-generation cephalosporin resistance from recurrence-free survival curves. Second, although the choice of our empiric antibiotic regimen followed the previous guidelines, variability in the regimen may have influenced the clinical outcomes, including recurrence [25,31]. However, variability in decision making for empiric antibiotics was low, and patients who had their therapy changed or those who were administered additional aminoglycosides were initially excluded from the cohort (*n* = 61, Figure 1). The empiric treatment protocol followed the current UTI guidelines, although they do not offer specific recommendations regarding the management of resistant UTIs [1]. Third, we included febrile hospitalized patients who had undergone VCUG; therefore, it is likely that patients with moderate to severe clinical manifestations who already had unknown risk factors were selected, resulting in high recurrence rates. Although VCUG was performed according to the guidelines, in which VCUG is recommended in recurrent febrile UTI cases, atypical clinical conditions, or when the renal US reveals hydronephrosis, scarring, or other findings that suggest obstructive uropathy, some patients had been evaluated using the “bottom-up approach” based on the clinicians’ decision, which would have mitigated and compensated for such bias.

In conclusion, third-generation cephalosporin resistance was a significant risk factor for early recurrence in pediatric patients with UTI who had undergone VCUG. Although the precise cause could not be clarified in this study, further treatment strategies, including aggressive antibiotic therapy or close follow-up, in selected high-risk patients can be suggested based on these results. Further studies with larger cohorts are required to validate our results and evaluate the different treatment strategies for high-risk patients.

## Figures and Tables

**Table 1:** Baseline demographics and clinical characteristics.

Variables	Category	All	UTI with Recurrence within 1 Year	UTI without Recurrence within 1 Year	Unadjusted Odds Ratios for 1-Year Recurrence	*p*-Value
Cases, *n* (%)		210 (100)	55 (26.2)	155 (73.8)		
Age, Median (years)		0.4 (0.3–0.7)	0.3 (0.1–0.5)	0.4 (0.3–0.7)	0.64 (0.38–1.06)	0.081
Category, *n* (%)	≤3 mo	103 (49.0)	33 (60.0)	70 (45.2)	3.78 (0.82–17.37)	0.088
	4–24 mo	89 (42.4)	20 (36.4)	69 (44.5)	2.32 (0.19–10.95)	0.288
	2–17 y	18 (8.6)	2 (3.6)	16 (10.3)	Reference	-
Sex, male		149 (71.0)	38 (69.1)	111 (71.6)	0.89 (0.45–1.73)	0.723
Previous history of antibiotics		27 (12.9)	10 (18.2)	17 (11.0)	1.80 (0.77–4.22)	0.174
Duration of fever, days	At admission	1.7 ± 1.8	1.8 ± 2.0	1.7 ± 1.7	1.05 (0.89–1.23)	0.576
	After admission	1.0 ± 0.9	0.9 ± 0.9	1.0 ± 0.9	0.97 (0.70–1.35)	0.850
	Total	2.7 ± 2.1	2.8 ± 2.1	2.6 ± 2.0	1.03 (0.89–1.19)	0.684
Fever over 39 °C		102 (48.6)	26 (47.3)	76 (49.0)	0.93 (0.50–1.73)	0.823
Length of stay, days		6.8 ± 2.2	6.9 ± 2.6	6.7 ± 2.1	1.03 (0.90–1.18)	0.625
Initial inflammatory markers	WBC * 10^3^/µL	1.7 ± 0.7	1.7 ± 0.6	1.8 ± 0.7	1.00 (1.00–1.00)	0.366
	Neutrophils (%)	55.1 ± 14.3	55.9 ± 14.0	54.9 ± 14.5	1.01 (0.98–1.03)	0.650
	Lymphocytes (%)	32.4 ± 13.0	31.4 ± 13.6	32.8 ± 12.7	0.99 (0.97–1.02)	0.478
	N/L ratio	2.8 ± 4.7	2.3 ± 1.6	3.0 ± 5.3	0.96 (0.88–1.05)	0.391
	Platelet * 10^3^/µL	428.9 ± 143.3	421.9 ± 169.5	437.6 ± 126.2	1.00 (0.99–1.00)	0.144
	Erythrocyte Sedimentation Rate (mm/h)	37.4 ± 23.2	41.1 ± 25.6	36.1 ± 22.3	1.10 (1.00–1.02)	0.183
	C-Reactive Protein (mg/L)	65.0 ± 56.7	70.7 ± 52.2	62.9 ± 58.3	1.00 (1.00–1.01)	0.385
Duration of pyuria ≥ 3 days		108 (51.4)	30 (54.5)	78 (50.3)	1.19 (0.64–2.20)	0.591
Resistance to 3rd cephalosporins		32 (15.2)	10 (18.2)	22 (14.2)	1.34 (0.59–3.05)	0.481
Imaging studies						
Kidney US	Acute pyelonephritis	82 (39.0)	26 (47.3)	56 (36.1)	1.59 (0.85–2.95)	0.147
	Hydronephrosis	108 (51.4)	31 (56.4)	77 (49.7)	1.31 (0.70–2.43)	0.395
DMSA renal scan	Cortical defect	148 (70.5)	42 (76.4)	106 (68.4)	1.49 (0.74–3.03)	0.267
Other urinary tract abnormalities *		15 (7.1)	8 (14.5)	7 (4.5)	3.60 (1.24–10.45)	0.019
VCUG	VUR grade ≥ 3	50 (23.8)	28 (50.9)	22 (14.2)	3.27 (3.13–12.56)	<0.001
Treatment with non-susceptible antibiotics in vitro		27 (12.9)	8 (14.5)	19 (12.3)	1.22 (0.50–2.97)	0.664

Data are presented as the mean ± standard deviation.* Other urinary tract abnormalities included duplex kidney (*n* = 5, 2.4%), ureteropelvic junction obstruction (*n* = 4, 1.9%), ureterocele (*n* = 3, 1.4%), horseshoe kidney (*n* = 1, 0.5%), renal agenesis (*n* = 1, 0.5%), and renal dysplasia (*n* = 1, 0.5%). WBC: white blood cell. N/L ratio: neutrophil/lymphocyte ratio. US: ultrasonography. DMSA renal scan: dimercaptosuccinic acid renal scan. VCUG: voiding cystourethrogram. VUR: vesicoureteral reflux.

**Table 2:** Risk factors for early and late recurrence of UTI.

Outcome	Variables	Univariable		Multivariable		Multivariable (Bootstrap Adjusted)	
		Odds Ratio (95% CI)	*p*-Value	Odds Ratio (95% CI)	*p*-Value	Odds Ratio (95% CI)	Relative Bias (%)
Early (90-day) recurrence							
	Sex, female	2.48 (1.12–5.46)	0.025	2.37 (1.03–5.44)	0.042	2.32 (0.88—6.13)	2.2
	Resistance to 3rd cephalosporins	2.93 (1.20–7.16)	0.019	2.79 (1.08–7.20)	0.034	2.71 (1.02–8.25)	3.0
	Other urinary tract abnormalities *	3.40 (1.07–10.77)	0.037	3.13 (0.90–10.86)	0.072	3.78 (0.57–11.64)	−16.4
	VUR grade ≥ 3 on VCUG	2.49 (1.10–5.62)	0.028	2.45 (1.04–5.81)	0.041	2.47 (1.07–5.97)	−0.7
Late (1-year) recurrence							
	Resistance to 3rd cephalosporins	1.34 (0.59–3.05)	0.481				
	Other urinary tract abnormalities *	3.60 (1.24–10.45)	0.019	3.14 (0.98–10.06)	0.054	3.14 (1.01–10.17)	<0.1
	VUR grade ≥ 3 on VCUG	6.27 (3.13–12.56)	<0.001	6.06 (3.00–12.25)	<0.001	6.01 (3.18–12.01)	0.6

* Other urinary tract abnormalities included duplex kidney (*n* = 5, 2.4%), ureteropelvic junction obstruction (*n* = 4, 1.9%), ureterocele (*n* = 3, 1.4%), horseshoe kidney (*n* = 1, 0.5%), renal agenesis (*n* = 1, 0.5%), and renal dysplasia (*n* = 1, 0.5%). VCUG: voiding cystourethrogram. VUR: vesicoureteral reflux.

## Data Availability

All data are available on request.

## Supplementary Materials

The following supporting information can be downloaded at: https://www.mdpi.com/article/10.3390/jpm12050773/s1, Table S1: Treatment profiles of the patients; Table S2: (a): Antibiotic susceptibilities in urinary isolates of the whole cohort; (b): Antibiotic susceptibilities in urinary isolates of patients with third-generation cephalosporin resistance; (c): Antibiotic susceptibilities in urinary isolates from the first episode of pediatric febrile UTI in patients with recurrence within 1-year; Table S3: Risk factors for 60-day recurrence; Table S4: Risk factors for 90-day recurrence; Table S5: Risk factors for 180-day recurrence; Table S6: Risk factors for 1-year recurrence.

## Abbreviations

CI—confidence interval; DMSA—dimercaptosuccinic acid; MDR—multidrug resistance; OR—odds ratio; US—ultrasonography; UTI—urinary tract infection; VCUG—voiding cystourethrogram; VUR—vesicoureteral reflux.
