# Supplementary material for: Impact of Third-Generation Cephalosporin Resistance on Recurrence in Children with Febrile Urinary Tract Infections
Source: J Pers Med. 2022 May 10;12(5):773. doi: 10.3390/jpm12050773 (PMC9144345; doi:10.3390/jpm12050773)
Supplement: Supplementary file 1 [file jpm-12-00773-s001.zip › jpm-1680260-supplementary.pdf]

Journal of Personalized Medicine

MDPI

Supplementary Table S1. Treatment profiles of the patients.

		Third-generation Cephalosporin-Resistant UTIs (*n* = 32) *n*, (%)	Third-generation Cephalosporin-Sensitive UTIs (*n* = 178) *n*, (%)	Total (*n* = 210) *n*, (%)
Parenteral therapy	Ampicillin-sulbactam	0 (0)	2 (1.1)	2 (1.0)
	Ampicillin-sulbactam with aminoglycoside	3 (9.4)	55 (30.9)	58 (27.6)
	Ampicillin-sulbactam with third-generation cephalosporins	2 (6.3)	21 (11.8)	23 (11.0)
	Third-generation cephalosporins	23 (71.9)	94 (52.8)	117(55.7)
	Third-generation cephalosporins with aminoglycoside	4 (12.5)	6 (3.4)	10 (4.8)
Discharge antibiotics	Amoxicillin-clavulanate	0 (0)	8 (4.5)	8 (3.8)
	Third-generation cephalosporins	19 (59.4)	156 (87.6)	175 (83.3)
	Trimethoprim-sulfamethoxazole	3 (9.4)	5 (2.8)	8 (3.8)
	1st or 2nd-generation cephalosporins	2 (6.3)	0 (0)	2 (1.0)
	None	8 (25.0)	9 (5.1)	17 (8.1)

Supplementary Table S2a. Antibiotic susceptibilities in urinary isolates of the entire cohort.

Antibiotics	Number Nonsusceptible/Number Tested (%)
Amikacin	1/210 (0.5)
Amoxicillin-clavulanate	48/209(23.0)
Ampicillin-sulbactam	102/194 (52.6)
Cefepime	25/210 (11.9)
Cefotaxime	32/210 (15.2)
Ciprofloxacin	27/210 (12.9)
Ertapenem	0/210 (0)
Gentamicin	42/210 (20.0)
Imipenem	0/210 (0)
Meropenem	0/210 (0)
Piperacillin-Tazobactam	9/210 (4.3)
Tobramycin	38/210 (18.1)
Trimethoprim-sulfamethoxazole	62/210 (29.5)
Multidrug resistance	49/210 (23.3)

Nonsusceptible defined as intermediate susceptibility or resistant.

Multidrug resistance defined as nonsusceptibility to 3 or more of the following classes: aminoglycosides (amikacin, gentamicin, tobramycin), fluoroquinolones (ciprofloxacin, levofloxacin), carbapenem (ertapenem, imipenem, meropenem), trimethoprim-sulfamethoxazole, nitrofurantoin, beta-lactam/beta-lactamase inhibitor combination (amoxicillin-clavulanate, ampicillin-sulbactam, piperacillin-tazobactam), cefepime.

Supplementary Table S2b. Antibiotic susceptibilities in urinary isolates of patients with third-generation cephalosporin resistance.

Antibiotics	Number Nonsusceptible/Number Tested (%)
Amikacin	1/32 (3.1)
Amoxicillin-clavulanate	15/31 (48.4)
Ampicillin-sulbactam	29/32 (90.6)
Cefepime	25/32 (78.1)
Cefotaxime	32/32 (100.0)
Ciprofloxacin	12/32 (37.5)
Ertapenem	0/26 (0)
Gentamicin	13/32 (40.6)
Imipenem	0/32 (0)
Meropenem	0/32 (0)
Piperacillin-Tazobactam	1/32 (3.1)
Tobramycin	14/32 (43.8)
Trimethoprim-sulfamethoxazole	21/32 (65.6)
Multidrug resistance	23/32 (71.9)

Nonsusceptible defined as intermediate susceptibility or resistant.

Multidrug resistance defined as nonsusceptibility to 3 or more of the following classes: aminoglycosides (amikacin, gentamicin, tobramycin), fluoroquinolones (ciprofloxacin, levofloxacin), carbapenem (ertapenem, imipenem, meropenem), trimethoprim-sulfamethoxazole, nitrofurantoin, beta-lactam/beta-lactamase inhibitor combination (amoxicillin-clavulanate, ampicillin-sulbactam, piperacillin-tazobactam), cefepime.

Supplementary Table S2c. Antibiotic susceptibilities in urinary isolates from the first episode of patients with recurrence within 1 year.

Antibiotic	Number Nonsusceptible/Number Tested
Amikacin	0/55 (0)
Amoxicillin-clavulanate	12/54 (22.2)
Ampicillin-sulbactam	28/55 (50.9)
Cefepime	7/55 (12.7)
Cefotaxime	10/55 (18.2)
Ciprofloxacin	11/55 (20.0)
Ertapenem	0/55 (0)
Gentamicin	14/55 (25.5)
Imipenem	0/55 (0)
Meropenem	0/55 (0)
Piperacillin-Tazobactam	1/55 (1.8)
Tobramycin	12/55 (21.8)
Trimethoprim-sulfamethoxazole	16/55 (29.1)
Multidrug resistance	14/55 (25.5)

Nonsusceptible defined as intermediate susceptibility or resistant.

Multidrug resistance defined as nonsusceptibility to 3 or more of the following classes: aminoglycosides (amikacin, gentamicin, tobramycin), fluoroquinolones (ciprofloxacin, levofloxacin), carbapenem (ertapenem, imipenem, meropenem), trimethoprim-sulfamethoxazole, nitrofurantoin, beta-lactam/beta-lactamase inhibitor combination (amoxicillin-clavulanate, ampicillin-sulbactam, piperacillin-tazobactam), cefepime.

Supplementary Table S3. Risk factors for 60-day recurrence.

Variables	Category	Cases	Control	Univariable			Multivariable			Multivariable (Bootstrap-Adjusted)		
				Odds Ratio	95% Confidence Interval	*p*-Value	Odds Ratio	95% Confidence Interval	*p*-Value	Odds Ratio	95% Confidence Interval	Bias (%)
		24 (11.4)	186 (88.6)									
Age, Median (years)		0.3 (0.1-0.4)	0.4 (0.3-0.7)	0.687	(0.345, 1.367)	0.285						
Category, *n* (%)	≤3mo	17 (70.8)	86 (46.2)	1.581	(0.333, 7.520)	0.565						
	4-24 mo	5 (20.8)	84 (45.2)	0.476	(0.085, 2.672)	0.399						
	2-17 y	2 (8.3)	16 (8.6)	Reference	-	-						
Sex, female		11 (45.8)	50 (26.9)	2.302	(0.968, 5.471)	0.059						
Previous history of antibiotics		7 (29.2)	20 (10.8)	3.418	(1.264, 9.244)	0.015	2.520	(0.819, 7.758)	0.107	2.366	(0.522, 11.012)	6.8
Duration of fever, days	At admission	1.6±1.7	1.7±1.8	0.977	(0.763, 1.250)	0.850						
	After admission	0.6±0.7	1.0±1.0	0.590	(0.318, 1.095)	0.095						
	Total	2.3±1.9	2.7±2.1	0.887	(0.693, 1.136)	0.344						
Fever over 39°C		10 (41.7)	92 (49.5)	0.730	(0.309, 1.726)	0.473						
Length of stay, days		6.5±2.1	6.9±2.3	0.925	(0.753, 1.136)	0.457						
Initial inflammatory markers	WBC * 10^3/μL	1.6±0.7	1.8±0.7	1.000	(1.000, 1.000)	0.184						
	Neutrophils (%)	57.1±13.3	54.9±14.5	1.011	(0.981, 1.041)	0.478						
	Lymphocytes (%)	32.0±12.6	32.5±13.0	0.997	(0.965, 1.031)	0.869						
	N/L ratio	2.4±1.9	2.9±4.9	0.971	(0.858, 1.099)	0.642						

	Platelet * 10^3/μL	431.6±159.2	433.7±136.2	0.998	(0.995, 1.001)	0.181						
	Erythrocyte Sedimentation Rate (mm/hr)	39.4±28.6	37.2±22.6	1.004	(0.985, 1.023)	0.678						
	C-Reactive Protein (mg/L)	71.8±59.0	64.1±56.5	1.002	(0.995, 1.009)	0.530						
Duration of pyuria	≥3 days	17 (70.8)	91 (48.9)	0.049	(1.004, 6.400)	0.049	2.190	(0.800, 5.993)	0.127	2.035	(0.658, 10.074)	9.4
Resistance to 3rd cephalosporins		9 (37.5)	23 (12.4)	4.252	(1.670, 10.827)	0.002	3.150	(1.090, 9.105)	0.034	3.412	(1.082, 9.143)	-7.0
Imaging studies												
Kidney US	Acute pyelonephritis	12 (50.0)	70 (37.6)	1.657	(0.706, 3.890)	0.246						
	Hydronephrosis	12 (50.0)	96 (51.6)	0.938	(0.401, 2.194)	0.882						
DMSA renal scan	Cortical defect	17 (70.8)	131 (70.4)	1.020	(0.400, 2.597)	0.967						
Other urinary tract abnormalities		4 (16.7)	11 (5.9)	3.182	(0.926, 10.933)	0.066						
VCUG	VUR grade ≥3	12 (50.0)	38 (20.4)	3.895	(1.622, 9.352)	0.002	3.934	(1.545, 10.015)	0.004	3.610	(1.284, 13.971)	6.3

Data are presented as the mean ± standard deviation.

*Other urinary tract abnormalities included duplex kidney (*n* = 5, 2.4%), ureteropelvic junction obstruction (*n* = 4, 1.9%), ureterocele (*n* = 3, 1.4%), horseshoe kidney (*n* = 1, 0.5%), renal agenesis (*n* = 1, 0.5%), and renal dysplasia (*n* = 1, 0.5%)

WBC : white blood cell

N/L ratio : neutrophil/lymphocyte ratio

US : Ultrasonography

DMSA renal scan : dimercaptosuccinic acid renal scan

VCUG : Voiding cystourethrogram

VUR : Vesicoureteral reflux

Supplementary Table S4. Risk factors for 90-day recurrence .

Variables	Category	Cases	Control	Univariable			Multivariable			Multivariable (Bootstrap-Adjusted)		
				Odds Ratio	95% Confidence Interval	*p*-Value	Odds Ratio	95% Confidence Interval	*p*-Value	Odds Ratio	95% Confidence Interval	Bias (%)
		30 (14.3)	180 (85.7)									
Age, Median (years)		0.3 (0.1-0.5)	0.4 (0.3-0.7)	0.760	(0.461, 1.252)	0.281						
Category, *n* (%)	≤3mo	19 (63.3)	84 (46.7)	1.810	(0.383, 8.543)	0.454						
	4-24 mo	9 (30.3)	80 (44.4)	0.900	(0.177, 4.564)	0.899						
	2-17 y	2 (6.7)	16 (8.9)	Reference	-	-						
Sex, female		14 (46.7)	47 (26.1)	2.476	(1.123, 5.459)	0.025	2.368	(1.030, 5.444)	0.042	2.323	(0.881, 6.129)	2.2
Previous history of antibiotics		7 (23.3)	20 (11.1)	2.435	(0.927, 6.393)	0.071						
Duration of fever, days	At admission	1.5±1.6	1.7±1.8	0.905	(0.700, 1.170)	0.447						
	After admission	0.9±0.9	1.0±1.0	0.854	(0.545, 1.337)	0.490						
	Total	2.3±1.7	2.7±2.1	0.895	(0.717, 1.116)	0.323						
Fever over 39°C		15 (50.0)	87 (48.3)	1.069	(0.493, 2.316)	0.866						
Length of stay, days		6.8±2.7	6.8±2.2	1.003	(0.844, 1.193)	0.970						
Initial inflammatory markers	WBC * 10^3/μL	1.7±0.7	1.8±0.7	1.000	(1.000, 1.000)	0.294						
	Neutrophils (%)	55.6±12.6	55.1±14.6	1.003	(0.976, 1.030)	0.842						
	Lymphocytes (%)	31.6±11.9	32.6±13.2	0.994	(0.965, 1.024)	0.697						
	N/L ratio	2.3±1.7	2.9±5.0	0.957	(0.843, 1.087)	0.502						
	Platelet * 10^3/μL	428.0±202.6	434.4±125.6	0.998	(0.995, 1.001)	0.180						
	Erythrocyte Sedimentation Rate (mm/hr)	44.1±27.4	36.3±22.4	1.014	(0.997, 1.030)	0.104						
	C-Reactive Protein (mg/L)	73.9±53.1	63.5±57.3	1.003	(0.997, 1.009)	0.353						

Duration of pyuria ≥3 days		20 (66.7)	88 (48.9)	2.091	(0.927, 4.716)	0.076						
Resistance to 3rd cephalosporins		9 (30.0)	23 (12.8)	2.925	(1.195, 7.160)	0.019	2.792	(1.083, 7.198)	0.034	2.708	(1.022, 8.248)	3.0
Imaging studies												
Kidney US	Acute pyelonephritis	15 (50.5)	67 (37.2)	1.687	(0.776, 3.668)	0.187						
	Hydronephrosis	18 (60.0)	90 (50.0)	1.500	(0.683, 3.294)	0.312						
DMSA renal scan	Cortical defect	22 (73.3)	126 (70.0)	1.179	(0.494, 2.812)	0.711						
Other urinary tract abnormalities		5 (16.7)	10 (5.6)	3.400	(1.074, 10.767)	0.037	3.134	(0.904, 10.859)	0.072	3.778	(0.573, 11.635)	-16.4
VCUG	VUR grade ≥3	12 (40.0)	38 (21.1)	2.491	(1.104, 5.619)	0.028	2.453	(1.035, 5.811)	0.041	2.468	(1.071, 5.966)	-0.7

Data are presented as the mean ± standard deviation.

*Other urinary tract abnormalities included duplex kidney (n = 5, 2.4%), ureteropelvic junction obstruction (n = 4, 1.9%), ureterocele (n = 3, 1.4%), horseshoe kidney (n = 1, 0.5%), renal agenesis (n = 1, 0.5%), and renal dysplasia (n = 1, 0.5%)

WBC : white blood cell

N/L ratio : neutrophil/lymphocyte ratio

US : Ultrasonography

DMSA renal scan : dimercaptosuccinic acid renal scan

VCUG : Voiding cystourethrogram

VUR : Vesicoureteral reflux

Supplementary Table S5. Risk factors for 180-day recurrence.

Variables	Category	Cases	Control	Univariable			Multivariable			Multivariable (Bootstrap-Adjusted)		
				Odds Ratio	95% Confidence Interval	*p*-Value	Odds Ratio	95% Confidence Interval	*p*-Value	Odds Ratio	95% Confidence Interval	Bias (%)
		50 (23.8)	160 (76.2)									
Age, Median (years)		0.3 (0.2-0.5)	0.4 (0.3-0.7)	0.668	(0.408, 1.094)	0.109						
Category, *n* (%)	≤3mo	29 (58.0)	74 (74.3)	3.135	(0.678, 14.499)	0.144						
	4-24 mo	19 (38.0)	70 (43.8)	2.171	(0.459, 10.281)	0.328						
	2-17 y	2 (4.0)	16 (10.0)	Reference	-	-						
Sex, female		15 (30.0)	46 (28.7)	1.062	(0.530, 2.128)	0.865						
Previous history of antibiotics		10 (20.0)	17 (10.6)	2.103	(0.893, 4.951)	0.089						
Duration of fever, days	At admission	1.7±1.7	1.7±1.8	1.000	(0.838, 1.192)	0.997						
	After admission	0.9±0.9	1.0±1.0	0.956	(0.677, 1.350)	0.798						
	Total	2.6±1.8	2.7±2.1	0.990	(0.847, 1.159)	0.904						
Fever over 39°C		24 (48.0)	78 (48.8)	0.970	(0.514, 1.832)	0.926						
Length of stay, days		7.0±2.7	6.8±2.1	1.042	(0.907, 1.198)	0.559						
Initial inflammatory markers	WBC * 10^3/μL	1.7±0.6	1.8±0.7	1.000	(1.000, 1.000)	0.255						
	Neutrophils (%)	54.4±13.7	55.4±14.5	0.995	(0.973, 1.017)	0.653						
	Lymphocytes (%)	32.7±13.6	32.3±12.8	1.002	(0.978, 1.027)	0.862						
	N/L ratio	2.2±1.6	3.0±5.3	0.938	(0.833, 1.055)	0.286						
	Platelet * 10^3/μL	415.9±170.9	438.9±127.0	0.998	(0.996, 1.000)	0.072						
	Erythrocyte Sedimentation Rate (mm/hr)	42.6±25.8	35.8±22.2	1.012	(0.998, 1.026)	0.082						
	C-Reactive Protein (mg/L)	69.3±50.9	63.5±58.5	1.002	(0.996, 1.007)	0.534						
Duration of pyuria ≥3 days		28 (56.0)	80 (50.0)	1.273	(0.672, 2.410)	0.459						

Resistance to 3rd cephalosporins		10 (20.0)	22 (13.8)	1.568	(0.686, 3.583)	0.286						
Imaging studies												
Kidney US	Acute pyelonephritis	24 (48.0)	58 (36.3)	1.623	(0.855, 3.084)	0.139						
	Hydronephrosis	28 (56.0)	80 (50.0)	1.273	(0.672, 2.410)	0.459						
DMSA renal scan	Cortical defect	37 (74.0)	111 (69.4)	1.256	(0.614, 2.570)	0.532						
Other urinary tract abnormalities		8 (16.0)	7 (4.4)	4.163	(1.428, 12.141)	0.009	3.681	(1.177, 11.505)	0.025	3.462	(1.369, 11.623)	4.7
VCUG	VUR grade ≥3	24 (48.0)	26 (16.3)	4.757	(2.372, 9.541)	<0.001	4.636	(2.274, 9.454)	<0.001	4.522	(2.368, 9.440)	0.6

Data are presented as the mean ± standard deviation.

*Other urinary tract abnormalities included duplex kidney (*n* = 5, 2.4%), ureteropelvic junction obstruction (*n* = 4, 1.9%), ureterocele (*n* = 3, 1.4%), horseshoe kidney (*n* = 1, 0.5%), renal agenesis (*n* = 1, 0.5%), and renal dysplasia (*n* = 1, 0.5%)

WBC : white blood cell

N/L ratio : neutrophil/lymphocyte ratio

US : Ultrasonography

DMSA renal scan : dimercaptosuccinic acid renal scan

VCUG : Voiding cystourethrogram

VUR : Vesicoureteral reflux

Supplementary Table S6. Risk factors for 1-year recurrence.

Variables	Category	Cases	Control	Univariable			Multivariable			Multivariable (Bootstrap-Adjusted)		
				Odds Ratio	95% Confidence Interval	*p*-Value	Odds Ratio	95% Confidence Interval	*p*-Value	Odds Ratio	95% Confidence Interval	Bias (%)
		55 (26.2)	155 (73.8)									
Age, Median (years)		0.3 (0.1-0.5)	0.4 (0.3-0.7)	0.635	(0.381, 1.057)	0.081						
Category, *n* (%)	≤3mo	33 (60.0)	70 (45.2)	3.771	(0.819, 17.367)	0.088						
	4-24 mo	20 (36.4)	69 (44.5)	2.319	(0.491, 10.947)	0.288						
	2-17 y	2 (3.6)	16 (10.3)	Reference	-	-						
Sex, female		17 (30.9)	44 (28.4)	1.129	(0.577, 2.206)	0.723						
Previous history of antibiotics		10 (18.2)	17 (11.0)	1.804	(0.771, 4.223)	0.174						
Duration of fever, days	At admission	1.8±2.0	1.7±1.7	1.048	(0.890, 1.233)	0.576						
	After admission	0.9±0.9	1.0±0.9	0.969	(0.695, 1.350)	0.850						
	Total	2.8±2.1	2.6±2.0	1.031	(0.890, 1.194)	0.684						
Fever over 39°C		26 (47.3)	76 (49.0)	0.932	(0.503, 1.725)	0.823						
Length of stay, days		6.9±2.6	6.7±2.1	1.034	(0.903, 1.184)	0.625						
Initial inflammatory markers	WBC * 10^3/μL	1.7±0.6	1.8±0.7	1.000	(1.000, 1.000)	0.366						
	Neutrophils (%)	55.9±14.0	54.9±14.5	1.005	(0.984, 1.027)	0.650						
	Lymphocytes (%)	31.4±13.6	32.8±12.7	0.991	(0.968, 1.015)	0.478						
	N/L ratio	2.3±1.6	3.0±5.3	0.961	(0.877, 1.053)	0.391						
	Platelet * 10^3/μL	421.9±169.5	437.6±126.2	0.998	(0.996, 1.001)	0.144						
	Erythrocyte Sedimentation Rate (mm/hr)	41.1±25.6	36.1±22.3	1.099	(0.996, 1.023)	0.183						
	C-Reactive Protein (mg/L)	70.7±52.2	62.9±58.3	1.002	(0.997, 1.008)	0.385						

Duration of pyuria ≥3 days		30 (54.5)	78 (50.3)	1.185	(0.639, 2.196)	0.591						
Resistance to 3rd cephalosporins		10 (18.2)	22 (14.2)	1.343	(0.591, 3.051)	0.481						
Imaging studies												
Kidney US	Acute pyelonephritis	26 (47.3)	56 (36.1)	1.585	(0.850, 2.954)	0.147						
	Hydronephrosis	31 (56.4)	77 (49.7)	1.308	(0.705, 2.430)	0.395						
DMSA renal scan	Cortical defect	42 (76.4)	106 (68.4)	1.493	(0.736, 3.032)	0.267						
Other urinary tract abnormalities		8 (14.5)	7 (4.5)	3.599	(1.239, 10.451)	0.019	3.143	(0.982, 10.062)	0.054	3.142	(1.095, 10.166)	<0.1
VCUG	VUR grade ≥3	28 (50.9)	22 (14.2)	6.269	(3.129, 12.561)	<0.001	6.063	(3.001, 12.250)	<0.001	6.007	(3.180, 12.013)	0.5

Data are presented as the mean ± standard deviation.

*Other urinary tract abnormalities included duplex kidney (n = 5, 2.4%), ureteropelvic junction obstruction (n = 4, 1.9%), ureterocele (n = 3, 1.4%), horseshoe kidney (n = 1, 0.5%), renal agenesis (n = 1, 0.5%), and renal dysplasia (n = 1, 0.5%)

WBC : white blood cell

N/L ratio : neutrophil/lymphocyte ratio

US : Ultrasonography

DMSA renal scan : dimercaptosuccinic acid renal scan

VCUG : Voiding cystourethrogram

VUR : Vesicoureteral reflux
